# Effects of *FGF2* on characteristics of ovine mammary epithelial cells and milk production traits in dairy sheep

**DOI:** 10.3389/fvets.2025.1709582

**Published:** 2026-01-12

**Authors:** Yuan Zhao, Mingna Li, Zhiyun Hao, Longjie Che, Chunyan Ren, Jiqing Wang, Rui Xu, Haixiong Hu, Mingjie Li, Yijuan Li, Yuxin Feng, Zike Wang

**Affiliations:** 1 Gansu Key Laboratory of Herbivorous Animals Biotechnology, College of Animal Science and Technology, Gansu Agricultural University, Lanzhou, China; 2 Gansu Animal Husbandry General Station, Lanzhou, China

**Keywords:** *FGF2*, SNP, OMECs, milk fat, sheep, milk protein

## Abstract

**Introduction:**

Fibroblast growth factor 2 (FGF2), a pleiotropic growth factor, promotes the elongation of mammary ducts and therefore contributes to the morphological development of mammary. It plays a crucial role in the development of mammary gland and lactation processes. However, the impact of *FGF2* on milk production traits in sheep remains unexplored.

**Methods:**

In the study, the effects of *FGF2* on the characteristics of ovine mammary epithelial cells (OMECs) were investigated. Meanwhile, penta-primer amplification refractory mutation system (PARMS) was used to look for single nucleotide polymorphisms (SNPs) in nine regions of ovine *FGF2*. Additionally, the associations of sequence variations in *FGF2* with variations in average daily milk yield and seven milk composition traits were also investigated in 449 Yuansheng Aite dairy sheep.

**Results:**

Among seven ovine tissues expressed by *FGF2*, it had the highest expression levels in liver and mammary gland tissue. Over-expressed of ovine *FGF2* increased the viability, proliferation number and ratio of OMECs, as well as triglyceride content of OMECs. Conversely, silencing of ovine *FGF2* produced the opposite effect to over-expression. A total of four novel SNPs were identified, with three located in intron 1 and one in intron 2. The genotypes and presence (or absence) of alleles from *FGF2* were found to affect milk production traits in sheep. Ewes with the genotype *AA* at c.282 + 11,288 G/A had higher milk protein percentage, milk fat percentage, dry matter content and ash content. The presence of allele G at the c.282 + 11,288 G/A locus were associated with milk production traits described above in sheep.

**Discussion:**

These findings indicate that *FGF2* may serve as a potential molecular marker for improving milk production traits in sheep.

## 1 Introduction

As a “new increment” in China’s dairy industry, sheep milk has gained widespread recognition and popularity due to its unique nutritional composition, excellent digestibility and high nutritional value. Internationally, it is acclaimed as the “king of milk” (1). Sheep milk had higher levels of milk protein, casein, calcium, certain trace elements, and conjugated linoleic acid compared to cow’s milk and goat’s milk (2). Additionally, it exhibits hypoallergenic properties (3). Moreover, as the primary source, sheep milk is crucial to survival, growth, and normal development of new born lambs (4). Although China’s sheep milk industry has been hugely development in recent years, overall milk yield remains low, and large-scale production has yet to be established. In this context, improving the quality and quantity of sheep milk remains a key research focus of China’s dairy industry (5). Milk production traits are economically important, yet the heritability of certain traits is relatively low (6). This low heritability makes traditional breeding methods time-consuming and inefficient. With the rapid development of molecular biology, molecular breeding techniques have been integrated into traditional breeding methods (7). Marker-assisted selection can effectively shorten the generation interval and improve selection accuracy for traits with low heritability, and sex-limited traits if effective molecular genetic markers can be identified. Single Nucleotide Polymorphisms (SNPs) have become widely used as DNA molecular markers (8), due to they were associated with variations in various traits, such as growth, reproduction, and milk production. In breeding of dairy livestock, some important SNPs have been identified and researched into association with milk performance traits.

Fibroblast growth factor 2 (*FGF2*) is a member of the FGF family and the gene is significantly associated with mammary gland development and milk performance. For example, under the influence of a specific protein kinase, FGF2 specifically bound to the receptor FGFR, and consequently promoted the formation of breast branching morphology (9–11). At the same time, *FGF2* promoted mitosis in human mammary epithelial cells, thereby enhancing cell proliferation (12). *FGF2* also participated in regulation of the viability, proliferation and cell cycle progression of, mammary epithelial cells in dairy cows (13) and humans (14). FGF*2* was expressed in lactating mammary gland of dairy cows, suggesting that *FGF2* was involved in mammary gland development (15). In this context, *FGF2* was chosen as a candidate gene for lactation traits in domestic animals.

To date researches into effect of *FGF2* on mammary gland development and lactation performance in domestic animals have only been performed in dairy cows. Specifically, association between *FGF2* SNPs with lactation performance and effect of *FGF2* on proliferation and viability of mammary epithelial cells were investigated in dairy cows. For instance, Wang et al. (16) reported that g.11646 A > G polymorphism in intron 1 of *FGF2* was significantly associated with milk fat content and somatic cell score of dairy cows. Brzakova et al. (17) further found that this SNP described above affected milk yield in dairy cows. The SNP rs41609100 of *FGF2* has been found to significantly correlate with the attachment of the anterior mammary gland and the position of the anterior teat in cows (18). A novel SNP g.11863 T > C in intron 1 of *FGF2* was associated with milk production traits of dairy cows (19). Meanwhile, SNPs of *FGF2* were screened in various species, including mice, rabbits (20), frogs (21), pigs (22), goats, and cows. However, it is currently unknown whether the above-mentioned SNPs exist in ovine *FGF2* and whether these SNPs have an influence on milk performance traits in sheep. Meanwhile, the tissue expression of *FGF2* has not been constructed, and the effect of *FGF2* on the milk fat percentage has also not been investigated in domestic animals. Accordingly in this study, the regulatory effects of *FGF2* on the viability, proliferation, and triglyceride content of ovine mammary epithelial cells (OMECs) were explored. Meanwhile, nucleotide sequence variations of ovine *FGF2* were screened using penta-primer amplification refractory mutation system (PARMS) and Sanger sequencing. We also investigate the tissue expression of *FGF2* and associations of variations in the gene with variations in milk yield and milk quality in 449 dairy sheep. These findings provide a theoretical foundation for identifying molecular genetic markers that regulate milk production traits in dairy sheep.

## 2 Materials and methods

### 2.1 Experimental animals and sample collection

All animal experiments were approved by the Animal Experiment Ethics Committee of Gansu Agricultural University (Approval number GSAU-ETH-AST-2023-029).

In Gansu Yuansheng Agriculture and Animal Husbandry Technology Co., Ltd., a total of 449 Yuansheng Aite dairy ewes sourced from a single farm with complete pedigree information about age, lambing number, and parity were selected. A total mixed ration (TMR) was formulated according to nutritional requirements of dairy sheep. The primary formulated feed consisted of corn and soybeans, while alfalfa and oat hay were as roughages and required trace elements were also incorporated. Each ewe was provided with about 3.3 kg/d TMR and had unrestricted access to water. Milking was carried out in the morning and in the afternoon of every day, respectively. About 5 mL of blood samples were collected from the jugular vein of each experimental ewe into vacuum tubes containing EDTA anticoagulant and then stored at −20 °C. During mid-lactation, 50 mL milk samples were collected from each ewe to determine milk fat percentage, milk protein percentage, lactose percentage, ash content, non-fat milk solids content, dry matter content, and acidity using UL40BC milk composition analyzer (Youchuang, Hangzhou, China). The daily milk yield of each ewe was measured using a Riavar 9JP-2×24 parallel milking machine (DeLaVal, TianJin, China).

Six separate healthy two-year-old ewes during the mid-lactation period were selected. After slaughter, three samples of the heart, liver, spleen, lung, kidney, ovary, and mammary gland were collected from each ewe. The tissues were immediately rinsed with alcohol and phosphate-buffered saline (PBS), and then preserved in liquid nitrogen for subsequent RNA extraction.

### 2.2 Total RNA extraction and reverse transcription- quantitative PCR

Total RNA was extracted from seven ovine tissues and OMECs using TRIzol reagent (Invitrogen, Carlsbad, CA, United States), and its concentration and purity were assessed using a NanoDrop 8,000 spectrophotometer (NanoDrop Technologies, Wilmington, NC, USA). Subsequently, cDNA was synthesized using the SweScript All-in-One RT SuperMix (Servicebio, Wuhan, China). RT-qPCR analysis was conducted in triplicate on an Applied Biosystems QuantStudio 6 Flex Real-time PCR System (Thermo Fisher Scientific, Waltham, MA, USA) using 2 × SYBR Green qPCR Master Mix (Servicebio, Wuhan, China). The relative expression levels of *FGF2* in different tissues were determined using the 2^-ΔΔCt^ method, with ovine *β-actin* serving as a reference gene. The information of primer sequences is provided in Table 1.

**Table 1:** Primer sequence information.

Primer name	Primer sequence (5ʹ-3ʹ)	Amplicon size/bp	Annealing temperature/°C	Region of amplification
*FGF2^a^*	F: CACTTTAAGGACCCCAAGCG R: GGTAACGGTTTGCACACACT	260	60°C	CDS
*FGF2^b^*	F: CGcgcgtGCTCCCCGCGCGGCTCCAG R: CGCctagaTCAGCTCTTAGCAGACATTGGA	522	60°C	CDS
*SREBP1*	F: ACAGCCCGGTCTTTGAGG R: CCCAGGACGGTGGTTGAT	200	60°C	CDS
*FASN*	F: GGGCTCCACCACCGTGTTCCA R: GCTCTGCTGGGCCTGCAGCTG	226	60°C	CDS
*FABP4*	F: AATACTGAGATGTCCTTC R: TTTATGGTGGTTGATTTC	140	60°C	CDS
*β-actin*	F: AGCCTTCCTTCCTGGGCATGGA R: GGAGCGAAACTGACACCC	113	60°C	CDS

The lowercase letters “cgcgt” and “ctaga” represent restriction sites of MluI and XbaI, respectively. The uppercase letters on the left side of the restriction sites indicate protective bases, while the letters on the right side represent specific primer. ^a^It is designed for detection of expression levels RT-qPCR. ^b^It is designed for the construction of an over-expression vector of fibroblast growth factor 2 (FGF2).

### 2.3 Construction of over-expression vectors and silencing vectors of ovine *FGF2*

Based on the CDS region sequences of ovine *FGF2* (NM_001009769.1) deposited in NCBI, two PCR primers were designed using Primer3.0 software and detailed primer information is presented in Table 1. MluI and XbaI (TakaRa, Beijing, China) restriction enzymes sites were incorporated into the primer sequences. The CDS region was amplified using cDNA extracted from mammary gland tissue. Subsequently, the CDS region of ovine *FGF2* was successfully ligated into the mammalian expression vector pcDNA3.1(+) using DNA ligase (TakaRa, Beijing, China), and the over-expression vector pcDNA3.1-*FGF2* was therefore constructed. The pcDNA3.1 vector was used as a negative control. Three pairs of silencing sequences of ovine *FGF2* (named si-*FGF2*-1, si-*FGF2*-2 and si-*FGF2*-3) and their negative control si-NC were synthesized by Suzhou Hongxun Biotechnology Co., Ltd. The primer sequences are shown in Table 2.

**Table 2:** The siRNA sequences of fibroblast growth factor 2 (*FGF2*).

Name	Forward (5ʹ-3ʹ)	Reverse (5ʹ-3ʹ)
si-*FGF2*-1	GAAGAUUACUAGCUUCUAAAUTT	AUUUAGAAGCUAGUAAUCUUCTT
si-*FGF2*-2	GAGCGACCCUCACAUCAAACUTT	AGUUUGAUGUGAGGGUCGCUCTT
si-*FGF2*-3	CCAAGCGGCUGUACUGCAAGATT	UCUUGCAGUACAGCCGCUUGGTT

### 2.4 Cell proliferation and viability

The OMECs used in the experiment were derived from frozen cell samples stored in Gansu Key Laboratory of Herbivorous Animals at College of Animal Science and Technology, Gansu Agricultural University. The purified OMECs were cultured in 24-well plates containing DMEM/F12 medium (Hyclone, UT, United States) in an incubator at 37 °C with 5% CO_2_. To investigate the effect of ovine *FGF2* on proliferation of OMECs when the confluence of OMECs reached 70 ~ 80%, pcDNA3.1-*FGF2*, pcDNA3.1, si-*FGF2*, si-NC were transfected into OMECs (n = 4), using the INVI DNA & RNA Transfection Reagent™ (Invigentech, CA, United States). At 44 h post-transfection, the CellLight™ EdU kit (Beyotime, Shanghai, China) was used to conduct an EdU assay. The staining results were visualized using a fluorescence microscope (IX73) (Olympus, Tokyo, Japan). During microscopic observation and imaging, five fields of view were randomly selected per well. The number of EdU-labeled proliferated OMECs was quantified using Image J software.

Meanwhile, after transfection of pcDNA3.1-*FGF2*, pcDNA3.1, si-*FGF2* and si-NC into OMECs for 44 h, 10 μL of CCK-8 reagent (Invigentech, CA, United States) was added to each well, and OMECs were incubated at 37 °C for an additional 4 h. The absorbance value of OMECs at 450 nm was subsequently measured using a Varioskan™ LUX microplate reader (Thermo Fisher Scientific, MA, USA). Three replications of each of the above experiments were performed.

### 2.5 Detection of triglyceride content

After transfection of pcDNA3.1-*FGF2*, pcDNA3.1, si-*FGF2* and si-NC into OMECs for 48 h (*n* = 4), the OMECs were digested with 0.25% trypsin (Gibco, Carlsbad, CA, United States), and then centrifuged at 1200 rpm for 5 min at 25 °C. A solution of n-heptane and isopropanol was prepared at 1:1 volume ratio, and designated as solution 1. A total of 1 mL of solution 1 was added to collected cell pellet. The mixture was subjected to ultrasonication for 1 min. Subsequently, the suspension was centrifuged at 8000 g for 10 min at 4 °C. The supernatant was collected for further analysis. The triglyceride content was quantified using a triglyceride content detection kit (Solarbio, Beijing, China) according to the manufacturer’s instructions, and then measured at 420 nm with a Varioskan™ LUX microplate reader (Thermo Fisher Scientific, MA, USA). Three replications of the above experiments were performed.

### 2.6 DNA extraction and PCR amplification

A total of 0.8 mL of blood sample from each ewe was dropped onto FTA cards (Whatman International Ltd., Maidstone, United Kingdom). Genomic DNA was extracted from blood collected on FTA cards using the two-step method described by Zhou et al. (23). Based on ovine *FGF2* sequences (Gene ID: 443306), nine sets of PCR primers were designed to amplify a part of intron 1, intron 2, exon 1, exon 2 and exon 3 using the Primer3.0 software. The primers were synthesized by Yangling Tianrun Aoke Biotechnology Co., Ltd. The detailed information of PCR primers is shown in Table 3.

**Table 3:** Primer sequence information for screening of SNPs in ovine fibroblast growth factor 2 (*FGF2*).

Primer name	Primer sequence (5ʹ-3ʹ)	Amplicon size/bp	Annealing temperature/°C	Region of amplification
Primer 1	F: AAGTGCTCTCCTTCAGTCCC R: TGTGGACTGAACTGTACCCC	422	60°C	Intron 1
Primer 3	F: ACTGGCATTCATCCCTAGGG R: ACCACGATACAGCACAGGAT	441	62°C	Intron 1
Primer 4	F: TGCAGTTTCCTTCTCTCCTCT R: GGTTTGAGATCAGAAGCAGAAGA	361	58°C	Intron 2
Primer 5	F: GCATCACCACGCTGCCAG R: ACTGGGGTCACACTGAAGAG	279	60°C	Exon 1
Primer 6	F: GTGAGAAGGGAGTCTGGAGG R: TCCATCTTCTTTCATAGCAAGGT	302	58°C	Exon 2
Primer 7	F: TGTTCCTGTCCTGCATCCTT R: AGGTCCTGTTTTGGGTCCAA	309	58°C	Exon 3
Primer 8	F: TGGATAACTACTCATGGGCCA R: AGAATGAGAGGACAATGCCCA	338	60°C	Intron 1
Primer 9	F: ACCAAGAAAACACAGCTCGT R: TCCATGGACAGAGGAGCCT	289	60°C	Intron 2

PCR amplification was performed using a 20 μL reaction system, containing one 1.2 mm DNA card, 0.8 μL each of the upper and downstream primers, 10.4 μL of 2 × Green Taq Master Mix (Thermo Fisher Scientific, MA, United States) and 8.0 μL of ddH2O. PCR reaction condition consisted of pre-denaturation at 94 °C for 5 min, denaturation at 94 °C for 30 s, annealing for 30 s (see Table 3 for annealing temperatures), and extension at 72 °C for 30 s, with a final extension of 10 min at 72 °C. PCR amplification results were checked by 1.5% agarose gel electrophoresis.

### 2.7 Sanger sequencing

A total of 40 samples were randomly selected from 449 ewes and then used for SNPs screening. The specific PCR products amplified were mixed in equal volumes and then sent to Tianrun Aoke Biotechnology Co., Ltd. for Sanger sequencing. Subsequently, the SNPs were identified using Chromas2.6.5 software.

### 2.8 PARMS genotyping and sequence analyses

The genomic DNA were samples extracted from FTA cards and its concentration and purity were assessed using a NanoDrop 8,000 spectrophotometer (NanoDrop Technologies, Wilmington, NC, United States). Samples with a concentration exceeding 20 ng/μL and a purity (A260/280) ranging from 1.8 to 2.0 were selected for the subsequent experiments. The SNPs loci screeded were genotyped using the PARMS method at Wuhan Jingtai Biotechnology Co., Ltd. Briefly, PCR amplifications were performed in a 20 μL reaction consisting of 2 μL genomic DNA template, 10 μL 2 × PARMS mix, 2 μL mixed primers (100 μmol/L), and 6 μL ddH₂O. Simultaneously, a blank well was established in each set of experiment as a negative control. Subsequently, fluorescence intensities of carboxyfluorescein (FAM) and hexachloro-fluorescein phosphoramidite (HEX) were measured at 518 nm and 559 nm, respectively, using a TECAN Infinite M1000 Microplate Reader (TECAN, Männedorf, Switzerland). Each set of experiment was conducted three times. Finally, scatter plots were generated to determine genotypes of ovine *FGF2* using Microsoft Excel 2024 software. Ultimately, a total of 449 samples were successfully genotyped, achieving a genotyping success rate of 93%. The sequences of four SNP-specific amplification primers are shown in Table 4. The Hardy–Weinberg equilibrium was tested using the chi-square (*χ*^2^) statistic.

**Table 4:** The information of genotyping primers using PARMS.

SNPs	Base	Primer name	Primer sequence (5ʹ-3ʹ)
SNP_1_	*T*	SNP_1_-Ft	GAAGGTGACCAAGTTCATGCTGGCCCACTCTATAACAGCCCT
	*C*	SNP_1_-Fc	GAAGGTCGGAGTCAACGGATTGCCCACTCTATAACAGCCCC
	-	SNP_1_-R	CAAATTCATACACTGAAGCCCG
SNP_2_	*C*	SNP_2_-Fc	GAAGGTGACCAAGTTCATGCTTGTAGTTAAAGAGCTGGAACACTCC
	*T*	SNP_2_-Ft	GAAGGTCGGAGTCAACGGATTGTGTAGTTAAAGAGCTGGAACACTCT
	-	SNP_2_-R	CACTGGGTGGAGAAGGGAAG
SNP_3_	*G*	SNP_3_-Fg	GAAGGTGACCAAGTTCATGCTGGAGGCAGTGTATATTTTTGCTG
	*A*	SNP_3_-Fa	GAAGGTCGGAGTCAACGGATTTGGAGGCAGTGTATATTTTTGCTA
	-	SNP_3_-R	TCCATTTCAAAGGTTTATTAACCTG
SNP_4_	*G*	SNP_4_-Fg	GAAGGTGACCAAGTTCATGCTCTCTAAAATATTAGCAGTTGGCTGAG
	*A*	SNP_4_-Fa	GAAGGTCGGAGTCAACGGATTCTCTAAAATATTAGCAGTTGGCTGAA
	-	SNP_4_-R	CAAGGGTGTCCCACCTATAATG

GAAGGTGACCAAGTTCATGCT represents the FAM-labeled fluorescent sequence, while GAAGGTCGGAGTCAACGGATT represents the HEX-labeled fluorescent sequence. The symbol F denotes the forward primer that recognizes different bases and R denotes the reverse universal primer.

### 2.9 Statistical analysis

SPSS16.0 and GraphPad Prism 8.02 were used to data analysis and image generation, respectively. Two-tailed independent t-tests were used for comparisons between the two groups, while one-way ANOVA was used for comparisons between multiple groups. The results are presented as mean ± standard errors of the mean (SEM).

For genotypes and alleles with a frequency >5% (thus providing adequate sample size), the general linear mixed-effects models (GLMMs) in SPSS16.0 were employed to investigate associations between *FGF2* common genotypes or the presence or absence of *FGF2* common alleles and variations in average daily milk yield and seven milk composition. A Bonferroni correlation was used to decrease the probability of false positive results during the multiple comparisons in the model. To identify the appropriate fixed and random effects in the model, we evaluated the influence of age, lambing number, and parity on lactation traits in sheep. The findings indicated that neither lambing number nor parity had a statistically significant effects on lactation traits, whereas age had a significant effect on lactation traits in the 449 ewes. Consequently, age was included in the GLMMs.

The statistical model was defined as: Y = *μ* + Genotype (Allele) + Age + e, where Y represents the lactation traits, μ denotes the population mean, Genotype refers to the specific genotype, Allele corresponds to the allele, Age indicates the age of ewe, and e represents the random residual error. In the model, genotype allele and age were fitted as fixed factors. Flock was not included in the model, as all 449 ewes came from a single farm. The confidence intervals were set at 95%, and the model assumptions were assessed, including normality, homoscedasticity, and independence.

## 3 Results

### 3.1 Expression of ovine *FGF2* in seven tissues

**Figure 1:**
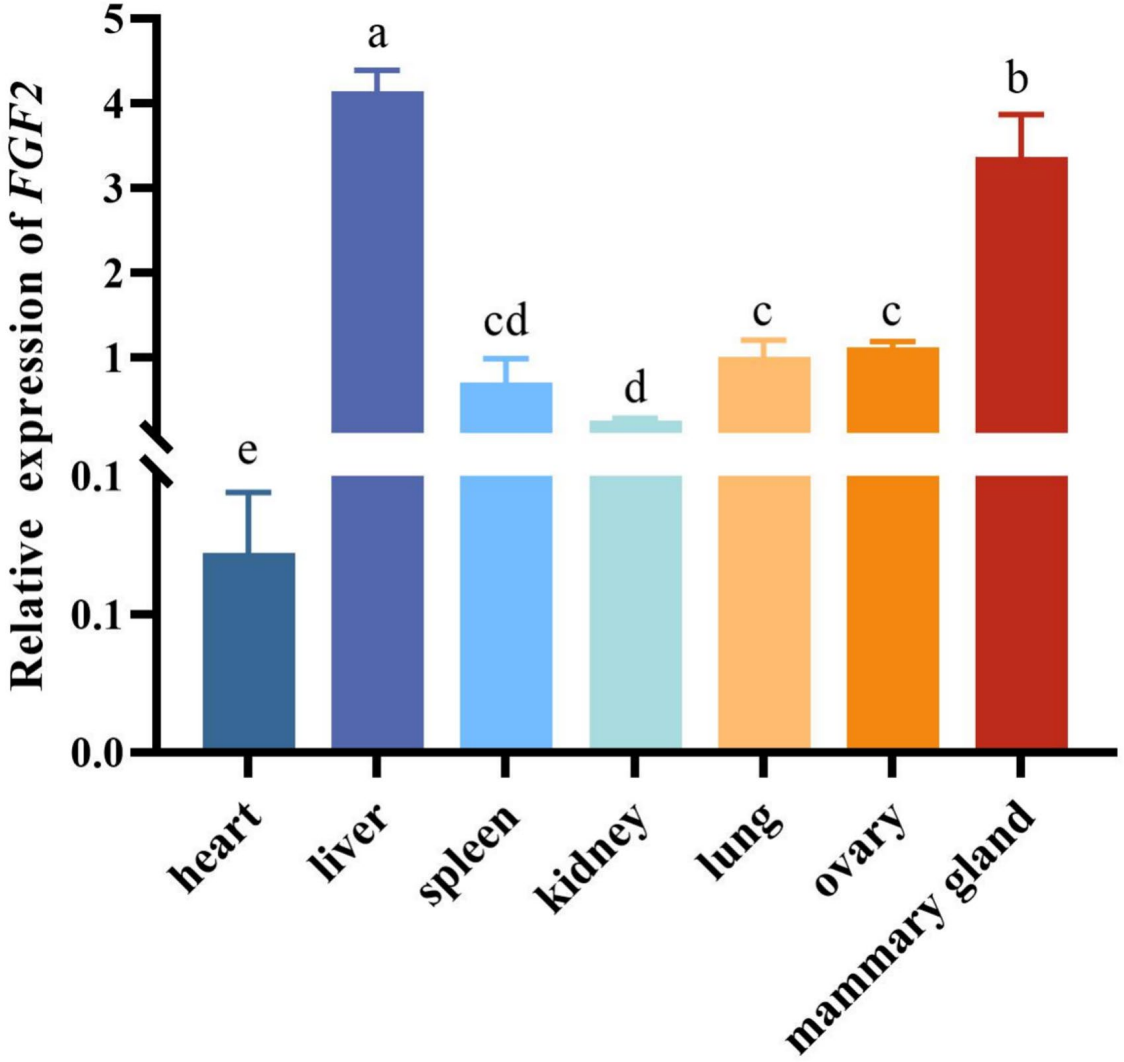
Relative expression levels of fibroblast growth factor 2 (*FGF*2) in ovine tissues. Different lowercase letters represent significant differences (*p* < 0.05).

The RT-qPCR analysis revealed that *FGF2* was expressed in seven different tissues of sheep. Specifically, the liver showed the highest expression level of *FGF2*, followed by the mammary gland. The gene had the lowest expression levels in the heart and kidney (Figure 1).

### 3.2 *FGF2* enhances the proliferation and viability of OMECs

**Figure 2:**
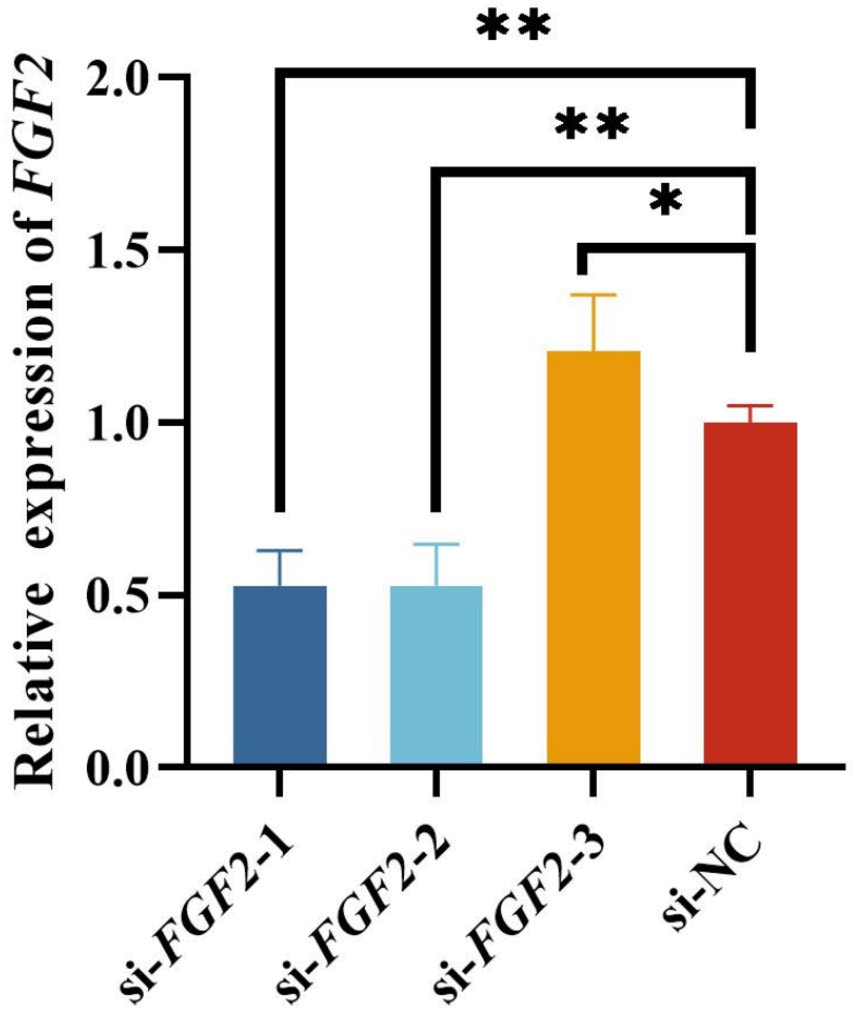
Assessment of silencing efficiency of ovine Fibroblast growth factor 2 (*FGF2*). *p < 0.05 and **p < 0.01.

The RT-qPCR analysis results demonstrated that si-*FGF2*-2 had the best inhibitory effect on *FGF2* expression compared to si-NC (Figure 2). Consequently, si-*FGF2*-2 was selected for subsequent experiments.

**Figure 3:**
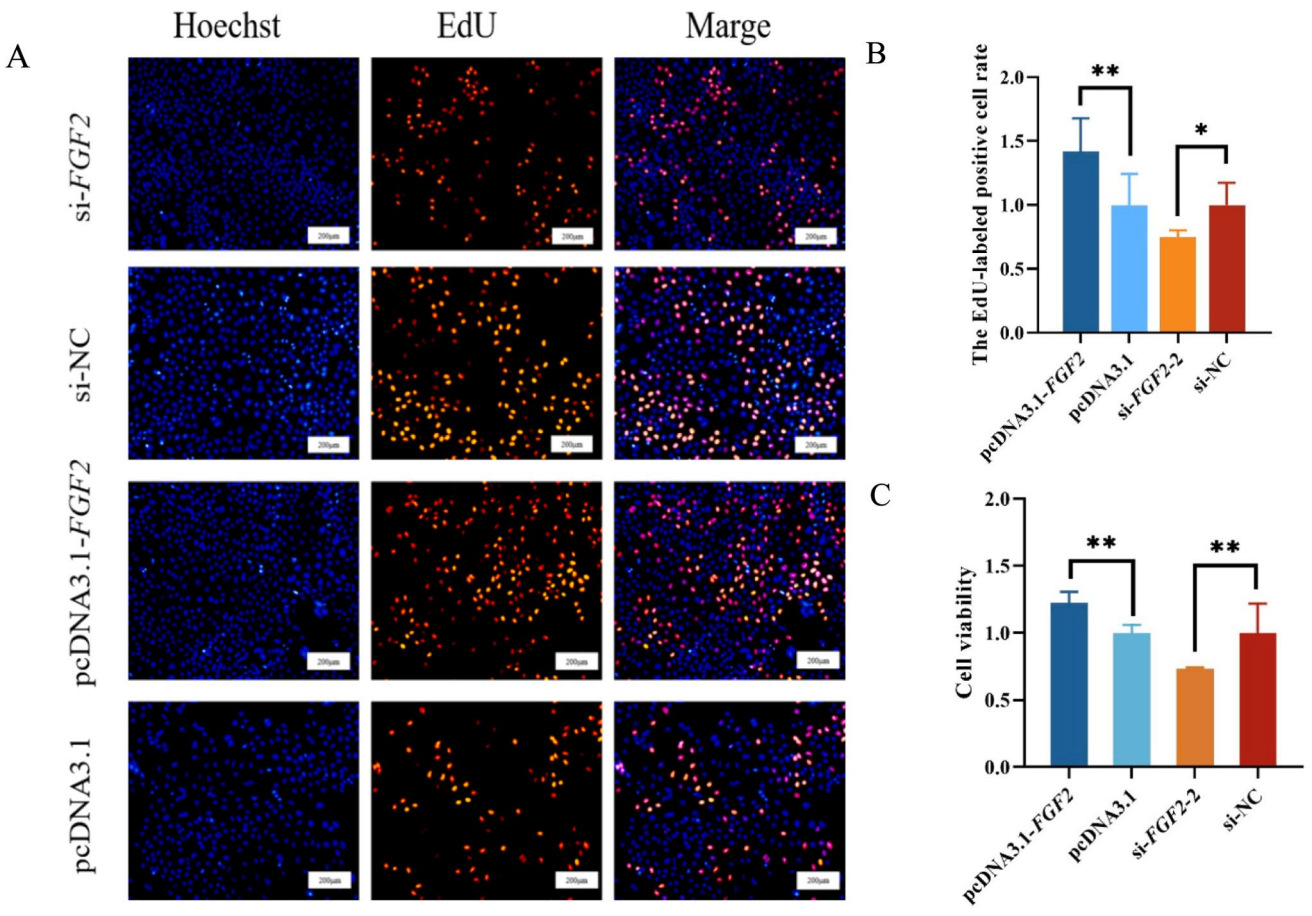
The effect of fibroblast growth factor 2 (*FGF2*) on the proliferation and viability of ovine mammary epithelial cells (OMECs). **(A)** Effect of *FGF2* on the number of proliferated OMECs. **(B)** The percentage of 5-ethynyl-2′-deoxyuridine (EdU)-labeled positive OMECs was calculated using ImageJ software. **(C)** Effect *FGF2* on the viability of OMECs, detected using a cell counting kit-8 (CCK-8) assay. *p < 0.05 and **p < 0.01. Scale bar indicates 200 μm; microscopic magnification: 200 ×.

The EdU assay results indicated that over-expressed *FGF2* significantly increased both the proliferation number (Figure 3A) and the proportion of positive OMECs (Figure 3B), whereas silencing of *FGF2* significantly suppressed OMECs proliferation (Figure 3A) and reduced the proportion of positive OMECs (Figure 3B). The CCK-8 assay results demonstrated that over-expression of *FGF2* led to a significant increase in cell viability compared to its negative control, while silencing of *FGF2* produced the opposite effect (Figure 3C).

### 3.3 *FGF2* increases the content of triglycerides

**Figure 4:**
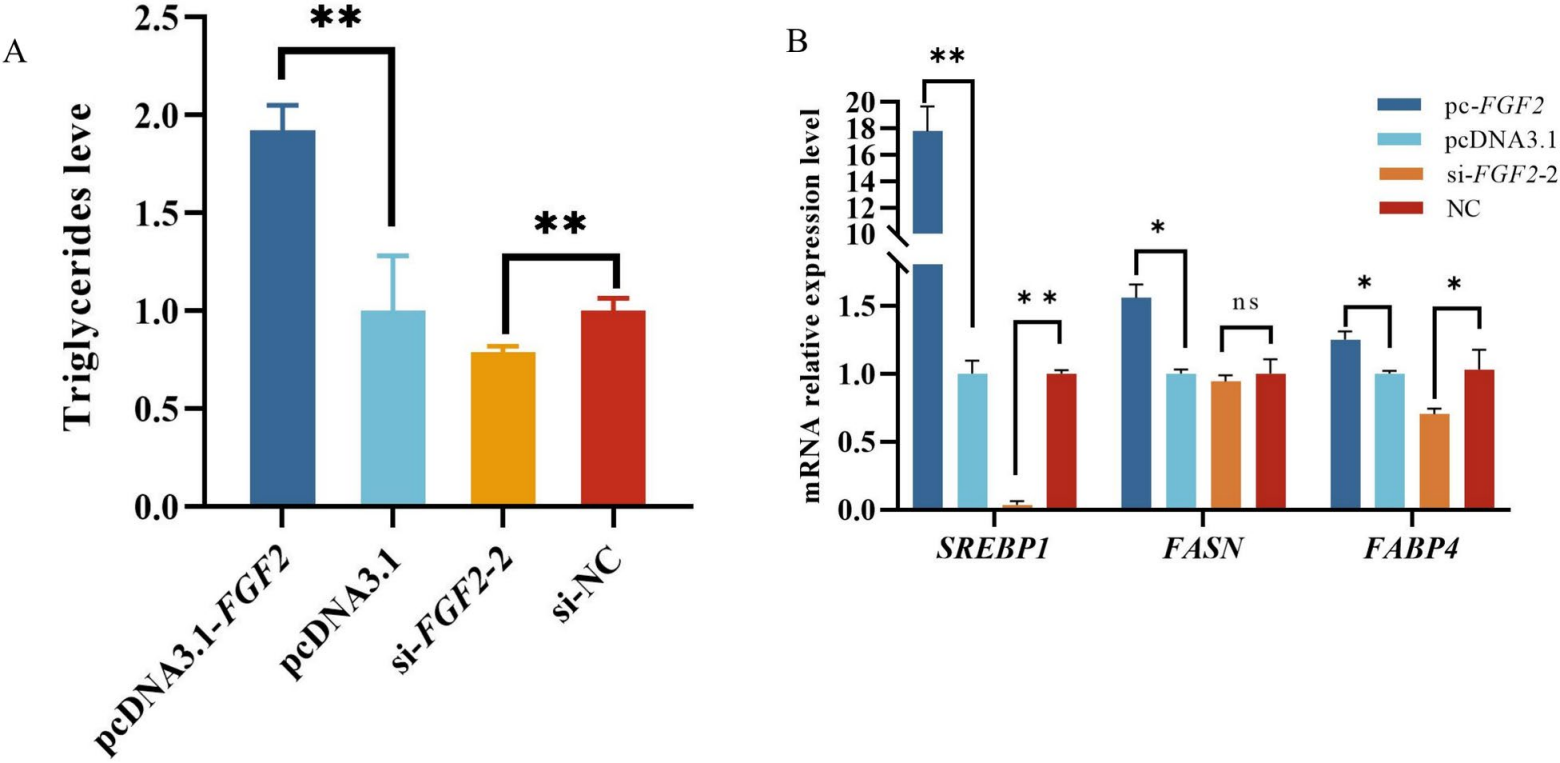
**(A)** Effect of ovine fibroblast growth factor 2 (*FGF2*) on triglyceride content in ovine mammary epithelial cells (OMECs). **(B)** The mRNA expression levels of genes associated with milk fat biosynthesis. Ns: not significant; *p < 0.01 and **p < 0.01.

Over-expressed *FGF2* significantly increased the triglyceride content of OMECs when compared with the pcDNA3.1. However, si-*FGF2* significantly decreased triglyceride content in OMECs (Figure 4A). Meanwhile, over-expression of the *FGF2* significantly enhanced the expression levels of milk fat biosynthesis related genes sterol regulatory element-binding protein1 (SREBP1), fatty acid synthase *(FASN)*, and fatty acid-binding protein 4 (FABP4), while silencing of *FGF2* markedly reduced the expression levels of *SREBP1* and *FABP4* (Figure 4B). These suggest that ovine *FGF2* increased the contents of triglycerides of OMECs.

### 3.4 Detection results of ovine *FGF2* SNPs

**Figure 5:**
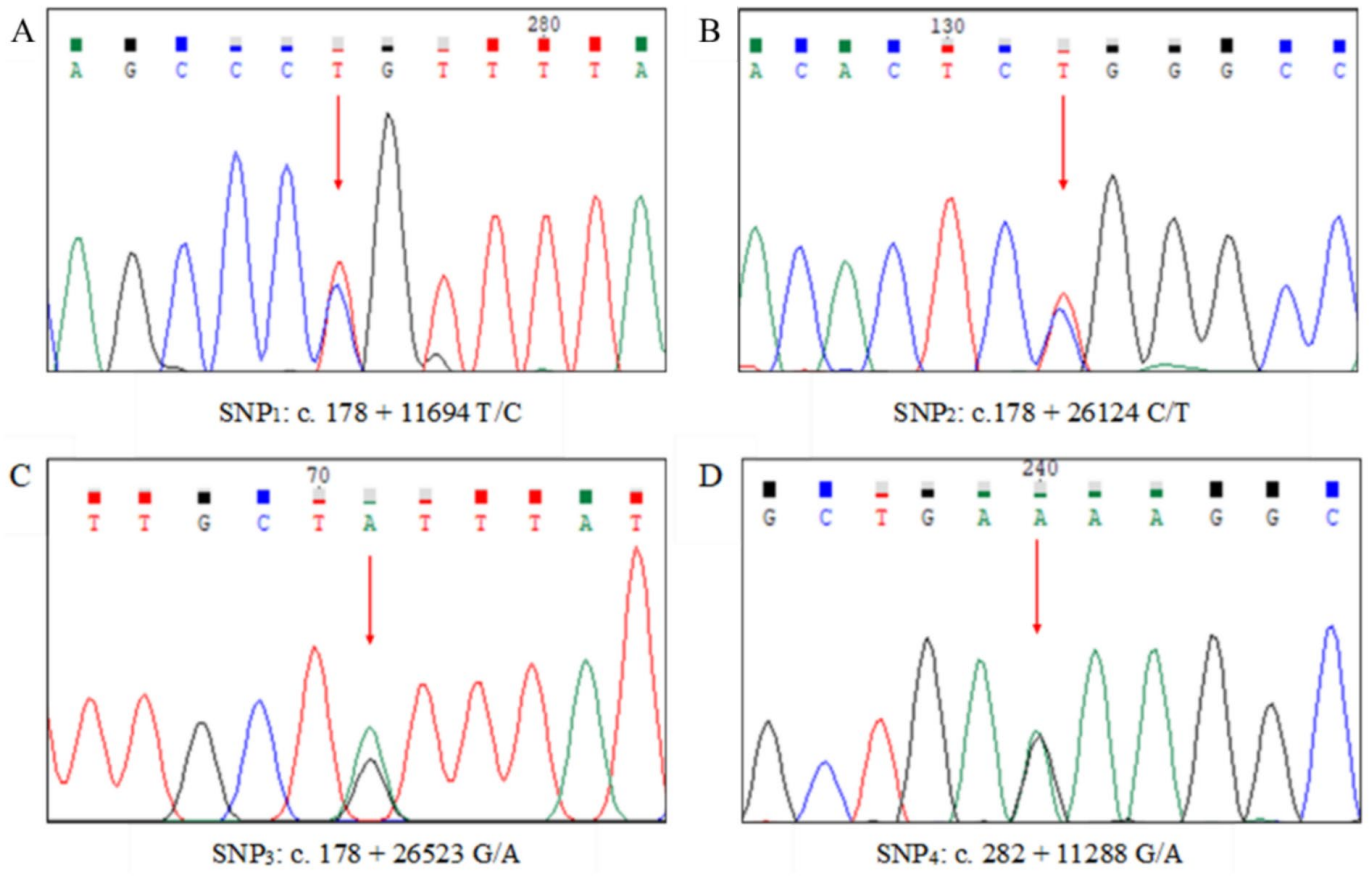
DNA sequence chromatograms of SNP_1_ **(A)**, SNP_2_ **(B)**, SNP_3_ **(C)**, and SNP_4_ **(D)** in ovine fibroblast growth factor 2 (*FGF2*). The red arrow represents the precise location of the SNPs on the DNA sequence chromatograms.

Of the nine regions of ovine *FGF2* detected, a total of four SNPs were identified, with three [c.178 + 11,694 T/C (SNP_1_), c.178 + 26,124C/T (SNP_2_)] and [c.178 + 26,523 G/A (SNP_3_)] found in intron 1 and one [c.282 + 11,288 G/A (SNP_4_)] located in intron 2 (Figure 5). No SNPs were detected in the other regions of *FGF2* amplified.

### 3.5 Genotyping results of ovine *FGF2* SNPs

**Figure 6:**
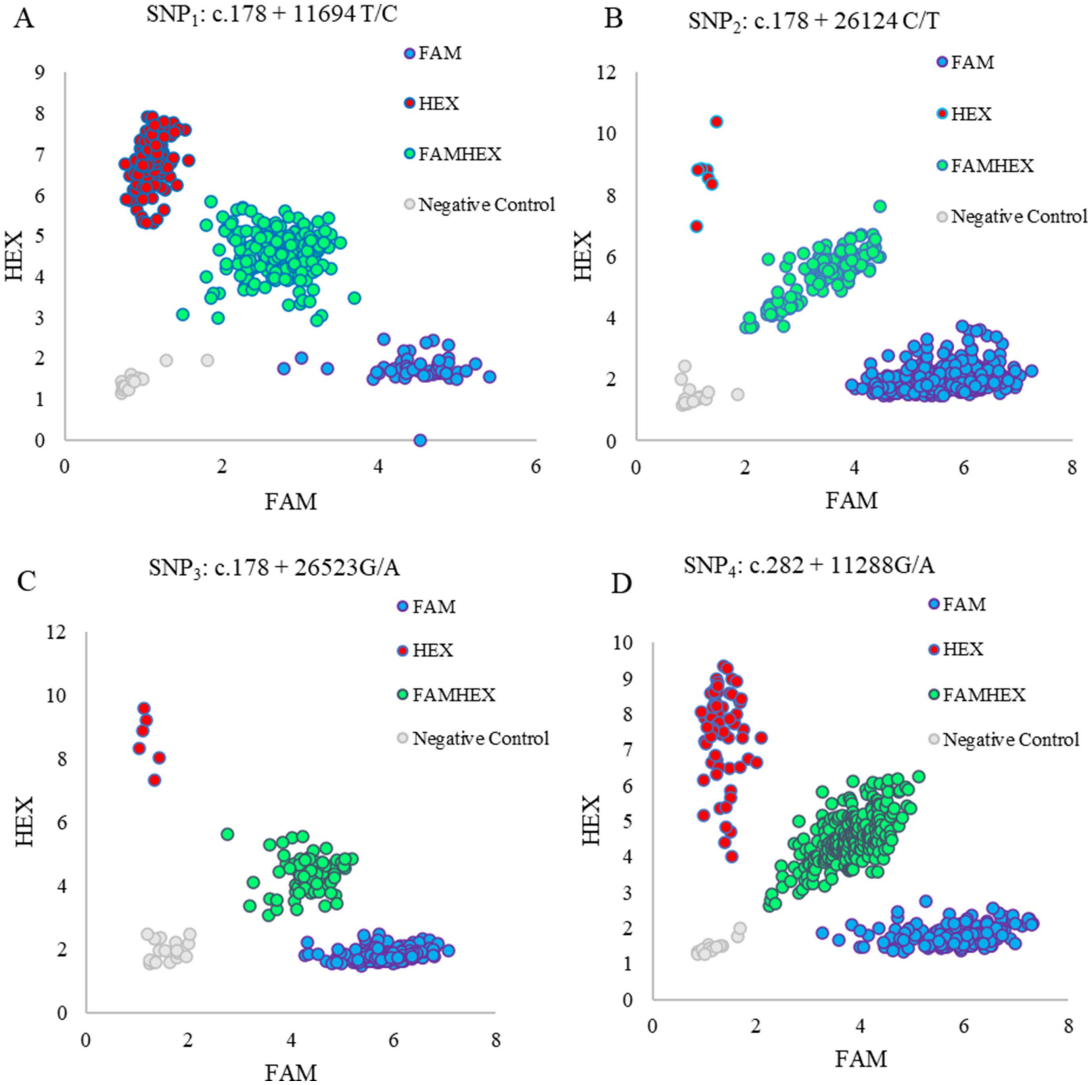
Genotyping results by PARMS of SNP_1_ **(A)**, SNP_2_ **(B)**, SNP_3_ **(C)**, and SNP_4_ **(D)** in ovine 585 fibroblast growth factor 2 (*FGF2*). FAM: Wild genotype; HEX: mutant genotype. FAMHEX: heterozygous genotype. Negative control: no DNA template added.

In each SNP locus, there were three genotypes. The genotypes *TT*, *TC*, and *CC* were defined in the SNP_1_ 178 + 11,694 T/C (Figure 6A), while the genotypes *TT*, *CT*, and *CC* were found in the SNP_2_ c.178 + 26,124C/T (Figure 6B). The SNP_3_ c.178 + 26,523 G/A presented genotypes *AA*, *GA*, and *GG* (Figure 6C), while the SNP_4_ c.282 + 11,288 G/A showed genotypes *GG*, *GA*, and *AA* (Figure 6D).

### 3.6 Genetic polymorphism analysis in ovine *FGF2*

Allele frequencies of ovine *FGF2* are summarized in Table 5. For SNP_1_ c.178 + 11,694 T/C, C was the most common allele with a frequency of 64.48%. For SNP_2_ c.178 + 26,124C/T, the frequencies of alleles Tand C were 15.14 and 84.86%, respectively. For SNP_3_ c.178 + 26,523 G/A, G was the most common allele with a frequency of 84.74%. In SNP_4_ c.282 + 11,288 G/A, the frequencies of alleles G and A were 60.13 and 39.87%, respectively. Chi-square tests revealed that all four SNPs were in Hardy–Weinberg equilibrium (*p* > 0.05).

**Table 5:** Allele frequencies of ovine fibroblast growth factor 2 (*FGF2*) in dairy sheep.

SNPs	Genotype frequencies (%)			Allele frequencies (%)		*p*-value
SNP_1_	*TT*	*TC*	*CC*	*T*	*C*	0.170
	11.13	48.78	40.09	35.52	64.48	
SNP_2_	*TT*	*TC*	*CC*	*T*	*C*	0.226
	1.56	27.17	71.27	15.14	84.86	
SNP_3_	*AA*	*GA*	*GG*	*A*	*G*	0.208
	1.56	27.39	71.05	15.26	84.74	
SNP_4_	*GG*	*GA*	*AA*	*G*	*A*	0.789
	35.86	48.55	15.59	60.13	39.87	

### 3.7 Effect of common genotypes in *FGF2* on milk production traits in sheep

The genotypes *TT* at SNP_2_ and *AA* at SNP_3_ were occurred at a frequency of less than 5%, so the association of the two genotypes with milk production traits was not investigated given this low frequency and potential for bias.

For the SNP_4_ c.282 + 11,288 G/A, ewes with the genotype *AA* had the highest milk protein percentage (*p* = 0.032), milk fat percentage (*p* = 0.011) dry matter content (*p* = 0.002) and ash content (*p* = 0.027), when compared to the genotypes *AG* and *GG*. No associations were found between genotypes from the SNP_1_ c.178 + 11,694 T/C, the SNP_2_ c.178 + 26,124C/T or the SNP_3_ c.178 + 26,523 G/A and any milk production traits (Table 6).

**Table 6:** Association of common genotypes of SNPs in ovine fibroblast growth factor 2 (*FGF2*) with milk production traits in sheep.

SNPs	Genotype	Genotype number	Non-fat milk solid content (%)	Milk protein percentage (%)	Milk fat percentage (%)	Average daily milk yield (kg/d)	Dry matter content (%)	Milk lactose percentage (%)	Acidity (°T)	Ash content (%)
SNP_1_	*CC*	180	11.488 ± 0.066	5.241 ± 0.055	5.268 ± 0.182	0.880 ± 0.028	16.821 ± 0.194	5.338 ± 0.006	17.745 ± 0.396	0.844 ± 0.006
	*TC*	219	11.408 ± 0.056	5.157 ± 0.046	5.050 ± 0.182	0.924 ± 0.023	16.542 ± 0.194	5.336 ± 0.005	18.119 ± 0.334	0.836 ± 0.005
	*TT*	50	11.611 ± 0.132	5.329 ± 0.110	4.663 ± 0.363	0.969 ± 0.055	16.339 ± 0.387	5.361 ± 0.012	18.617 ± 0.790	0.853 ± 0.011
	*p* value		0.394	0.336	0.359	0.339	0.468	0.210	0.621	0.370
	*η^2^*		0.013	0.016	0.015	0.015	0.011	0.022	0.007	0.014
SNP_2_	*CC*	320	11.493 ± 0.041	5.236 ± 0.034	5.165 ± 0.113	0.914 ± 0.017	16.723 ± 0.121	5.340 ± 0.004	17.994 ± 0.248	0.844 ± 0.003
	*CT*	122	11.370 ± 0.082	5.133 ± 0.068	5.076 ± 0.223	0.912 ± 0.035	16.512 ± 0.238	5.334 ± 0.008	18.055 ± 0.489	0.833 ± 0.007
	*p* value		0.488	0.498	0.219	0.966	0.251	0.469	0.950	0.455
	*η^2^*		0.010	0.020	0.022	0.001	0.010	0.011	0.001	0.011
SNP_3_	*GG*	319	11.496 ± 0.041	5.239 ± 0.034	5.164 ± 0.113	0.915 ± 0.017	16.725 ± 0.120	5.340 ± 0.004	18.004 ± 0.247	0.844 ± 0.003
	*GA*	123	11.363 ± 0.081	5.127 ± 0.067	5.080 ± 0.221	0.910 ± 0.034	16.508 ± 0.236	5.333 ± 0.008	18.030 ± 0.484	0.832 ± 0.007
	*p* value		0.424	0.429	0.220	0.960	0.246	0.432	0.954	0.390
	*η^2^*		0.012	0.012	0.022	0.001	0.020	0.012	0.001	0.013
SNP_4_	*AA*	70	11.646 ± 0.108	5.387 ± 0.089^a^	5.962 ± 0.234^a^	0.940 ± 0.046	17.673 ± 0.309^a^	5.335 ± 0.010	18.560 ± 0.655	0.860 ± 0.009^a^
	*GA*	218	11.360 ± 0.057	5.118 ± 0.047^c^	4.988 ± 0.155^b^	0.876 ± 0.024	16.413 ± 0.163^b^	5.333 ± 0.005	18.083 ± 0.345	0.832 ± 0.005^c^
	*GG*	161	11.522 ± 0.072	5.255 ± 0.060^b^	4.875 ± 0.196^b^	0.949 ± 0.031	16.462 ± 0.206^b^	5.349 ± 0.007	17.748 ± 0.437	0.845 ± 0.006^b^
	*p* value		0.062	**0.032**	**0.011**	0.228	**0.002**	0.293	0.644	**0.027**
	*η^2^*		0.039	0.048	0.063	0.021	0.083	0.018	0.006	0.051

Different lowercase letters represent significant differences (*p* < 0.05), while η^2^ denotes the magnitude of the effect size. The bold values indicate *P* < 0.05.

### 3.8 The effect of *FGF2* alleles on milk production traits in sheep

Because the frequency of ewes without allele C at SNP_2_ and the frequency of ewes without allele G at SNP_3_ were all 0%, the two alleles were excluded from the association with milk production traits for the same reasons as above.

For the SNP_4_ c.282 + 11,288 G/A, the presence of G was associated with decreased milk protein percentage (present: 5.176 ± 0.027; absent: 5.399 ± 0.090; *p* = 0.030), milk fat percentage (present: 4.941 ± 0.089; absent: 5.952 ± 0.292; *p* = 0.003), dry matter content (present: 16.434 ± 0.094; absent: 17.667 ± 0.307; *p* < 0.001), and ash content (present: 0.838 ± 0.003; absent: 0.861 ± 0.009; *p* = 0.024; Table 7).

**Table 7:** Effect of ovine fibroblast growth factor 2 (*FGF2*) common allele on milk production traits in sheep.

SNPs	Allele assessed	Absent/present	Number	Non-fat milk solid content (%)	Milk protein percentage (%)	Milk fat percentage (%)	Average daily milk yield (kg/d)	Dry matter content (%)	Milk lactose percentage (%)	Acidity (°T)	Ash content (%)
SNP_1_	T	Present	269	11.448 ± 0.067	5.190 ± 0.039	4.975 ± 0.130	0.933 ± 0.020	16.488 ± 0.138	5.341 ± 0.004	18.216 ± 0.281	0.840 ± 0.004
	T	Absent	180	11.485 ± 0.067	5.239 ± 0.055	5.274 ± 0.182	0.880 ± 0.028	16.824 ± 0.193	5.337 ± 0.006	17.738 ± 0.394	0.843 ± 0.006
	*p* value			0.705	0.553	0.271	0.195	0.244	0.689	0.302	0.637
	*η^2^*			0.001	0.003	0.009	0.012	0.010	0.001	0.005	0.002
	C	Present	339	11.444 ± 0.032	5.194 ± 0.027	5.146 ± 0.088	0.905 ± 0.014	16.655 ± 0.094	5.337 ± 0.003	17.954 ± 0.192	0.839 ± 0.003
	C	Absent	50	11.609 ± 0.132	5.327 ± 0.110	4.659 ± 0.363	0.970 ± 0.055	16.334 ± 0.387	5.361 ± 0.012	18.624 ± 0.788	0.853 ± 0.011
	*p* value			0.258	0.271	0.225	0.287	0.453	0.078	0.442	0.265
	*η^2^*			0.009	0.009	0.010	0.008	0.004	0.022	0.004	0.009
SNP_2_	T	Present	129	11.337 ± 0.080	5.137 ± 0.066	4.994 ± 0.219	0.910 ± 0.034	16.437 ± 0.233	5.336 ± 0.007	18.087 ± 0.476	0.833 ± 0.007
	T	Absent	320	11.496 ± 0.041	5.238 ± 0.034	5.130 ± 0.112	0.913 ± 0.017	16.901 ± 0.119	5.341 ± 0.004	18.007 ± 0.243	0.844 ± 0.003
	*p* value			0.257	0.245	0.638	0.932	0.407	0.647	0.899	0.211
	*η^2^*			0.009	0.010	0.002	0	0.005	0.001	0	0.011
SNP_3_	A	Present	130	11.370 ± 0.079	5.130 ± 0.065	4.998 ± 0.217	0.908 ± 0.033	16.434 ± 0.231	5.335 ± 0.007	18.061 ± 0.471	0.833 ± 0.007
	A	Absent	319	11.499 ± 0.041	5.241 ± 0.034	5.128 ± 0.112	0.914 ± 0.017	16.693 ± 0.119	5.341 ± 0.004	18.017 ± 0.242	0.844 ± 0.003
	*p* value			0.212	0.200	0.650	0.883	0.393	0.557	0.943	0.171
	*η^2^*			0.011	0.012	0.001	0	0.005	0.002	0	0.013
SNP_4_	G	Present	379	11.428 ± 0.033^b^	5.176 ± 0.027^b^	4.941 ± 0.089^b^	0.907 ± 0.014	16.434 ± 0.094^b^	5.340 ± 0.003	17.942 ± 0.198	0.838 ± 0.003^b^
	G	Absent	70	11.659 ± 0.108^a^	5.399 ± 0.090^a^	5.952 ± 0.292^a^	0.946 ± 0.046	17.677 ± 0.307^a^	5.336 ± 0.010	18.532 ± 0.651	0.861 ± 0.009^a^
	*p* value			0.063	**0.030**	**0.003**	0.457	**<0.001**	0.739	0.427	**0.024**
	*η^2^*			0.024	0.062	0.033	0.004	0.083	0.001	0.005	0.036
	A	Present	288	11.429 ± 0.049	5.184 ± 0.040	5.224 ± 0.133	0.891 ± 0.020	16.719 ± 0.142	5.334 ± 0.004	18.199 ± 0.289	0.839 ± 0.004
	A	Absent	161	11.519 ± 0.073	5.253 ± 0.061	4.865 ± 0.201	0.948 ± 0.031	16.449 ± 0.214	5.439 ± 0.007	17.742 ± 0.436	0.845 ± 0.006
	*p* value			0.407	0.441	0.225	0.207	0.393	0.118	0.477	0.478
	*η^2^*			0.005	0.004	0.001	0.011	0.005	0.017	0.004	0.004

Different lowercase letters represent significant differences (*p* < 0.05), while η^2^ denotes the magnitude of the effect size. The bold values indicate *P* < 0.05.

No associations with any milk production traits were detected for the presence (or absence) of alleles in the SNP_1_ c.178 + 11,694 T/C, SNP_2_ c.178 + 26,124C/T, and A SNP_3_ c.178 + 26,523 G/A (Table 7).

## 4 Discussion

To date, there have been no reports documenting the expression profiles of *FGF2* across various tissues in domestic animals. Our RT-qPCR results indicated that *FGF2* had the highest levels in liver and mammary gland tissues. As a critical gene for fat metabolism and synthesis, *FGF2* suppressed thermogenic gene expression and promoted adipogenesis of mice (24). Additionally, in mouse models of fatty liver disease, *FGF2* has been found to accelerate hepatocyte proliferation (25). The high expression of *FGF2* in mammary gland tissues is inseparable from its function. *FGF2* played a crucial role in the branching morphogenesis of mouse mammary ducts and mammary tissue development (9). By binding to its receptor on mammary epithelial cells, *FGF2* activated signaling pathways that promote epithelial cell proliferation (13).

In the study, we constructed an overexpression vector for *FGF2* to transform its expression level beyond the normal physiological range. The results demonstrated that ovine *FGF2* promoted proliferation of OMECs and enhanced cellular viability. The findings were identical with other results in bovine mammary epithelial cells (13) and various types of cells in the body, including brain neural epithelial cells (26, 27), bone marrow stromal cells (28), vascular smooth muscle cells (29), and trophoblast cells (30). In human breast epithelial cells, *FGF2* functions as an autocrine factor, and disruption of this autocrine loop inhibited the proliferation of breast epithelial cells (31). This result implies a close correlation of *FGF2* and mammary gland cells proliferation. OMECs reside within the alveoli of the lobules and are the sole cell type responsible for milk production in mammary gland. It is therefore widely recognized that the viability and proliferation of OMECs directly influence milk yield and milk quality (32, 33). In this context, it was speculated that *FGF2* promoting the viability and proliferation of OMECs may influence milk production traits of sheep. However, this speculation was confirmed by our subsequent correlation analysis.

As the primary component of milk fat, triglycerides serve as an indicator for evaluating milk fat quality (34). Our results show that ovine *FGF2* increased triglyceride contents of OMECs. Meanwhile, *FGF2* can promote the expression of milk fat synthesis related genes *SREBP1*, *FASN*, and *FABP4*. However, to date, there have been no reports on effect of *FGF2* on triglyceride content of mammary epithelial cells. But *FGF2* has long been recognized for its association with the formation of adipose tissue (35). Additionally, an inhibitor of *FGF2* has been shown to prevent obesity and hepatic steatosis (24). In mammary gland tissue, *FGF2* secreted from visceral adipose tissue promoted the transformation of mammary epithelial cells (36). Furthermore, *SREBP1*, *FASN*, and *FABP4* were related to increased milk fat synthesis. For example, silencing of the forkhead box O1 (*FoxO1*) enhanced the expression levels of *FASN*, stearoyl-coA desaturase1 (*SCD1*), diacylglycerol acyltransferase1 (*DGAT1*), and *SREBP1*, thereby regulating fatty acid synthesis in mammary epithelial cells of dairy goats (37). It can be concluded that *FGF2* may promote triglyceride content in OMECs by regulating the expression of genes associated with milk fat synthesis.

Studies have demonstrated that both insulin-like growth factor1 (*IGF1*) and *FGF10* play crucial roles in mammary gland development. *IGF1* has been shown to promote bovine mammary gland growth (38), whereas *FGF10* enhanced the proliferation of mammary epithelial cells and suppressed apoptosis in mice (39). Similarly, *FGF2* has been shown to promote the proliferation of OMECs in the study. Although no studies have directly demonstrated the effects of *IGF1* and *FGF10* on milk fat synthesis, Rong et al. (40) demonstrated that *IGF1* promoted fat deposition, which may lead to obesity in humans. Furthermore, studies in goats have shown that *FGF10* enhanced intramuscular fat deposition. These suggest that *FGF2* had similar effect to *IGF1* and *FGF10*.

The ovine *FGF2* located on chromosome 17 includes three exons and two introns, and its full length is 54,589 bp. In this study, based on data from the SheepVar database and previous research findings in dairy cows (16, 18, 19), we screened nucleotide variations in nine amplification regions spanning 3,152 bp that contain exon 1, intron 1, exon 2, intron 2, and exon 3 in a sample of 449 ewes. Four SNPs were detected in intron, while no SNPs were found in exon regions. These SNPs found in the study were identified for the first time in sheep. Up to now, the research into nucleotide sequence variations of *FGF2* bas only been limited in dairy cows. A total of four SNPs g.11474 (C/G), g.11513 (C/G), g.11646 (A/G), and g.11863 (T/C) were found within intron 1 of *FGF2* in Holstein dairy cows and g.11646 (A/G) was significantly associated with milk yield, somatic cell count, and milk fat percentage (17, 19, 41). However, none of the four SNPs described above were detected in the dairy sheep population investigated in this study. This may be related to interspecific difference of *FGF2* sequences.

Although four SNPs identified in this study were located in intron regions of ovine *FGF2*, they were significantly associated with milk performance traits based on association analysis. The SNPs in intron may exert biological effects through multiple mechanisms. Jo and Choi et al. (42) demonstrated that introns can modulate gene expression levels by promoting alternative splicing. Furthermore, introns may modulate transcription initiation by affecting the function of the promoter of a gene. The regulatory function of introns is closely linked to their position. Studies have shown that the first intron is enriched in transcription factor binding motifs (43) and active histone modifications such as H3K4me1 and H3K4me3, which are positively correlated with gene expression levels (44).

In the study, the genotype *AA* at c.282 + 11,288 G/A had the highest milk fat percentage, milk protein percentage and dry matter and ash content. The percentages of milk protein, milk fat, dry matter content, and ash content in sheep’s milk were positively correlated (45), and the correlation explains why variations in ovine *FGF2* simultaneously affected the milk production performance. Milk protein, milk fat, dry matter and ash are important factors affecting milk quality. The protein and fat contents in goat milk directly influence cheese yield (46). The rich milk fat content is also a crucial factor contributing to the distinctive flavor of cheese (47). Casein is an important component for cheese formation and its high content facilitates cheese formation and maintains its shape (48). Ash primarily comprises major minerals such as calcium, phosphorus, sodium, potassium, magnesium, and trace elements. These minerals in sheep milk were essential for growth, bone development, and various biological functions (49). The dry matter content serves as an essential indicator for evaluating the nutritional and economic value of dairy products (50). These suggest that variations in ovine *FGF2* that affected the milk quality traits described above in the study had important breeding value. At SNP_4_ locus, the genotype *AA* had a 21.47% higher fat percentage than the genotype *GG*. The absence of the allele G decreased milk fat percentage by 18.64% compared to the presence of the allele G. In this context, selecting the ewes with the genotype *AA* or ewes with missing the allele G could result in increased economic benefits and this approach might therefore have some value for improving milk quality traits in sheep. Furthermore, as a growth factor, *FGF2* not only promoted mammary gland development, but also played a significant role in embryonic development (51) and growth traits (52). These suggest pleiotropic effects of *FGF2*.

## 5 Conclusion

The expression of *FGF2* exhibited tissue-specific in sheep. The *FGF2* enhanced the proliferation and cellular viability of OMECs, and also increased the content of triglycerides in OMECs. In addition, four novel SNPs were identified, with three located in intron 1 and one in intron 2. The genotype *AA* at c.282 + 11,288 G/A increased milk fat percentage, milk protein percentage, dry matter content, and ash content. The presence of allele G at c.282 + 11,288 G/A was also related to increased milk performance traits described above in sheep. However, the molecular mechanism by which c.282 + 11,288 G/A regulates milk production performance is still unknown, and further study is necessary to elucidate the mechanism in future. Meanwhile, effect of c.282 + 11,288 G/A on milk production performance needs to be confirmed in other sheep breeds.

## Data Availability

The original contributions presented in the study are included in the article/supplementary material, further inquiries can be directed to the corresponding author/s.
